# "The Hospital" and "The Globe"

**Published:** 1886-12-18

**Authors:** 


### "THE HOSPITAL" AND "THE GLOBE."

### LORD SALISBURY'S ARTICLE.

WE are reluctantly compelled by the statement in *The Globe* of the 18th inst. to call attention once more to this subject. Adhering to our determination not to characterise the transaction in question, we yet feel it to be due to our readers, the public, and the press to republish in parallel columns what we actually said, and what *The Globe* makes us say, as an interesting chapter in a little drama, which the *Pall Mall Gazette* has happily entitled "Behind the Scenes at *The Globe*":—

THE HOSPITAL,
*December 11th*, 1886.

THE matter (*i.e.* the premature publication of the article), has been submitted to counsel, the whole of the evidence has been thoroughly sifted, and we are advised that "the course adopted by the proprietors of *The Globe* is a good ground for an action for damages against them." In ordinary circumstances a writ would, of course, be immediately issued to recover compensation for the injury done to this Journal. The mission of THE HOSPITAL is, however, wholly peaceful and philanthropic, and its conductors prefer to take the more consistent course of standing by their principles, by refraining from retaliating on *The Globe*, notwithstanding the aggravating circumstance that not one word of apology or explanation has yet been published by the conductors of that paper. The publication of the following documents prepared by the solicitor and counsel of this Journal, will afford the Press and the public full facilities for passing judgment on a transaction which we prefer not to characterise. The following are the facts with regard to the premature and unauthorised publication of this article in *The Globe* of Wednesday, the 24th November, and, excepting the portion printed in italics, they are admitted by *The Globe* to be correct. The correspondence with *The Globe* submitted to counsel, affords the proof of the facts, and the printer's letter is proof of that portion of them disputed by *The Globe*.

THE GLOBE, *Dec. 10th*, 1886.

SOME few of our readers may possibly have seen the wholly unwarrantable charges against this journal made by the conductors of THE HOSPITAL in reference to the publication in our columns on the 24th ult. of a portion of Lord Salisbury's article "On Hospitals." Their account of the circumstances under which we were furnished with a copy of THE HOSPITAL is wholly inaccurate, so that the charge of "devices and misrepresentations" is absolutely groundless. We have given THE HOSPITAL every "explanation" of the way in which we received the paper from their recognised printer and publisher; but as to "apology," we, having regard to our own self-respect, have none to offer. The publication in our columns was intended as a tribute to the value of the article, and by way of compliment to the journal publishing it, as we at the time acknowledged that it was taken from THE HOSPITAL. Yet those who have seen THE HOSPITAL this week (of 11th inst.), will notice that our version of the transaction is not accepted. Disregarding, then, all suggestions as to "reparation," and all threats of legal proceedings—now that the conductors of that journal have chosen to make public their alleged grievances—we deem it due to ourselves to make this denial. We rest content, as we have already intimated, to await the decision of any tribunal that may be appealed to, preferring this course to troubling the public with correspondence of a contentious character.

